# Are nurses utilizing the non-pharmacological pain management techniques in surgical wards?

**DOI:** 10.1371/journal.pone.0258668

**Published:** 2021-10-21

**Authors:** Magda Mohamed Mohamed Bayoumi, Leena Mohammad Abdulla Khonji, Wessam Fathy Mohamed Gabr

**Affiliations:** 1 Department of Medical Surgical Nursing, Faculty of Nursing, Beni-Suef University, Beni Suef, Egypt; 2 Nursing Department, College of Health and Sport Sciences, University of Bahrain, Zallaq, Bahrain; Istanbul University Istanbul Faculty of Medicine: Istanbul Universitesi Istanbul Tip Fakultesi, TURKEY

## Abstract

The non-pharmacological pain management therapies have a valuable effect in managing moderate to mild pain intensity, especially if demonstrated in the pre-operative phase. The study aimed to explore the nurses’ practice toward using non-pharmacological pain management techniques in surgical wards. In a cross-sectional research design, a convenient sample of 47 nurses in the surgical wards in Egyptian hospital (Third Level) participated in the study. Data gathered using modified Non-pharmacological Methods Questionnaire. Results of the study indicated that nurse’s perception regarding applying the cognitive-behavioral methods as a distraction and Positive reinforcement techniques were more common (68.1%,53.2%), whereas most of them used emotional support (93.6%) and preferred to demonstrate physical methods. Meanwhile, nurses addressed the barriers to apply nonpharmacological pain management as lack of time, patient unwillingness, and patients’ health beliefs. Nevertheless, nurses reported the non-pharmacological pain management is less expensive and has fewer side effects than medication and can demonstrated post-discharge. Nurses play a key role in applying effective and different non-pharmacological therapies in surgical wards. Thus, nurses should be encouraged to demonstrate the non-pharmacological pain management therapies with patients undergoing surgical procedures.

## Introduction

Pain is a complex phenomenon including both the peripheral and central nervous systems that can impact a patient’s status physically, psychologically, and socially. Pain is defined by the American Pain Society (2008) as “an unpleasant sensory, and emotional experience associated with acute or potential tissue damage or described in terms of such damage” [1].

After procedures, approximately 20% of patients experience severe pain, particularly after surgery, for up to 24 hours, and this may extend throughout the wound healing process to 3 months [2].

Pain assessment is a challenge for nurses because it depends on the patient’s experience and is subjective rather than objective in nature [3]. Moreover, Pasero et al, (2009), reported that an estimated percentage of patients who complain of different levels of pain are not manageable, around 79% of hospitalized patients related to the gap between pain assessment and pain management [4].

Evidently, the achievement of optimal pain relief with both pharmacological as safe, effective analgesia and non-pharmacological pain management [5]. Non-pharmacological pain management techniques are usually effective for mild to moderate pain intensity, but they do not replace pharmacological pain therapies in patients with severe pain intensity [6]. Therefore, many patients report that the implementation of non-pharmacological methods can be helpful in coping and managing pain [7].

The effectiveness of the non-pharmacological management techniques is unpredictable, and the impact of relieving pain is different based on patients’ health beliefs, type of non-pharmacological method, time of application, and the intensity and duration of pain [8].

Nonpharmacological therapies are certainly less expensive and more applicable, as many alternative strategies, such as cognitive-behavioral therapy, physical therapy, emotional therapy, and patient family involvement, can be used [9–12].

The reduction of pain intensity is the main core responsibility of health care providers [13]. Indeed, nurses play a key role in providing care for patients undergoing surgery, while applying non-pharmacological pain management therapies. Thus, the nurses’ knowledge and attitude have a valuable effect in utilizing non-pharmacological pain management therapies [14]. Hence, this study aims to investigate the rate of utilizing Non-pharmacological Pain Management Techniques among nurses in surgical wards in Egypt.

## Materials and methods

### Design

The cross-sectional descriptive research design was used from December 2020 to January 2021.

### Setting and sample

A convenience sample of all nurses working in the surgical wards (n = 47) from third-level hospital, El-Mansura University Hospital, Egypt.

Inclusion criteria:

- Both sexes.
- All levels of education (diploma, technical institute, baccalaureate).
- All nurses caring for patients undergoing minor and major surgery.

Exclusion Criteria:

- Less than one year of experience.
- Unwilling nurses to participate.

### Data collection

The instrument in the study included 37 questions divided into 18 demographic and background questions and 19 questions about the Non-pharmacological Methods Questionnaire "Adapted from Tarja Polkki’s Nonpharmacological Methods Questionnaire, 2001 and modified by Bicek, 2004, to be more applicable to all patients, not just children" [15, 16]. Validity and reliability were tested for the original instrument with Cronbach’s alpha coefficient of 0.93, reflecting the internal consistency of the tool. The tool is divided into 3 parts as follows:

Part I: Demographic Questions included 5 questions (age, sex, level of education, years of experience, and Ward/Unit).
Part II: Nurses’ General pain Practices: 13 Questions about (amount of pain education, source of knowledge about pain, the practice of non-pharmacological pain management therapies without a doctor’s order, to how extent using the therapies).
Part III: Nonpharmacological Methods Questionnaire: 19 questions followed the Likert scale ranging from 1- "not at all" to 5- "always". The Questionnaire is divided into categories of nonpharmacological methods, including cognitive-behavioral methods (Qs19-27), physical methods (Qs28-30), emotional methods, and comfortable environment (Qs 31–33), and patient family involvement (Qs 36–37).

### Data analysis

Data was analyzed using the IBM SPSS software package version 20 (Armonk, NY: IBM Corp). Qualitative data was described using numbers and percentages. The Kolmogorov-Smirnov test was used to verify the normality of the distribution. Quantitative data was described using range (minimum and maximum), mean and standard deviation. The significance of the obtained results was judged at the 5% level. The used tests were Student t-test (for normally distributed quantitative variables, to compare between two studied groups), F-test (ANOVA) (for normally distributed quantitative variables, to compare between more than two groups), and univariate linear regression (to detect the most independent/affecting factor affecting overall non-pharmacological methods).

### Ethical considerations

Official letters were obtained from the Faculty of Nursing, Beni-Suef University to the surgical and nursing directors of the study settings, with Ethical Committee Approval # (N911-20). Informed consent obtained from volunteer participants after offering them a participant information sheet. All potential participants ensured the provision of confidentiality and anonymity. The researcher has also assured the administration that the conduction of the study will not affect the work in the study settings. Researchers disclosed the intention to disseminate the study findings once the study completed.

## Results and discussion

The demographic characteristics of nurses in the study sample are described in "Table 1." Nurses’ age ranged between 20 and 49 years, with a mean± SD (28.98 ± 7.30) years. More than a third quarter were females (80.9%) with a level of nursing certificate was mostly from technical institutes (44.72%) or baccalaureate (38.06%) levels. The years of experience ranged from novice to expert with a mean± SD of 9.36 ± 7.50 years.

**Table 1:** Distribution of the studied nurses according to demographic questionnaire (n = 47).

Q	Demographic Questionnaire	(N)	%
**1**	**Sex**		
	Male	9	19.1
	Female	38	80.9
**2**	**Age range (years)**		
	20–29	31	66.0
	30–39	11	23.4
	40–49	5	10.6
	Mean ± SD.	28.98 ± 7.30	
**3**	**Highest Level of education**		
	Diploma	6	17.22
	Technical institute	20	44.72
	Baccalaureate	16	38.06
**4**	**Years of experience**		
	1–9	34	72.3
	10–19	5	10.6
	20–29	8	17.0
	Mean ± SD.	9.36 ± 7.50	
**5**	Wards/Unit		
	Surgical wards	47	100%

Regarding the nurses’ general pain management practices," Table 2" illustrates that 51.1% of the study sample did not receive sessions about education, and more than one-third received up to 5 hours (42.6%) of educational pain sessions. Moreover, more than two-thirds of the study sample learned enough information on pain during the basic nursing education journey, and pain knowledge gained since graduation (76.6%). Meanwhile, thirty-one nurses received non-pharmacological classes of less than 5 hours, and the remaining nurses wished to learn about non-pharmacological pain management. Unfortunately, no tool was available for the assessment of pain, whereas only seven nurses mentioned that there were two tools for assessing pain: the happy-sad faces Tool (71.4%) and the visual analog scale (18.6%). However, six nurses were using the tool (85.7%).

**Table 2:** Distribution of the studied nurses according to nurses’ general pain practices (n = 47).

Q	Nurses’ general pain practices	(N)	%
**6**	**Amount of pain education in last 2 years**		
	None	24	51.1
	0–5 hours	20	42.6
	5–10 hours	3	6.4
**7**	**Did you feel you learned enough information on pain in school**		
	Yes	30	63.8
	No	17	36.2
**8**	**Is most of your knowledge about pain from**		
	Nursing practice since graduation	36	76.6
	Formal nursing school education	11	23.4
**9**	**Have you had any classes in non-pharmacological pain management in either nursing school or continuing education since graduation?**		
	Yes	31	66.0
	No	16	34.0
**10**	**If you answered yes to number 9, about how many hours did you have? (n = 31)**		
	0–5	24	77.4
	5–10	2	6.5
	10–15	4	12.9
	More than15	1	3.2
**12**	**If you answered no to number 9, do you wish you had more education involving non pharmacological pain management? (n = 16)**		
	Yes	14	87.5
	No	2	12.5
**13**	**Is there a pain assessment tool available for evaluating patient’s pain on your unit?**		
	No	40	85.1
	**Yes, what**	**7**	14.9
	**If yes (n = 7)**		
	Happy-sad face	5	71.4
	Visual analogue scale	2	28.6
**14**	**If you answered yes to number 13, do you use the tool? (n = 7)**		
	Yes	6	85.7
	No	1	14.3

Table 3 describes the non-pharmacological pain techniques that may be utilized without doctor order ". It is evident that the cognitive method that had the highest score was distraction, followed by positive reinforcement, relaxation, and breathing techniques (68.1%, 53.2%, 36.2%, and 36.2% respectively). While nurses can apply the physical method, a massage, followed by thermal regulation (cold/heat) positioning (29.8%, 21.3%, and 17.0% respectively). Conversely, emotional support is reflected at the highest level among all non-pharmacological techniques (93.6%). Additionally, nurses reported the demonstration rate of non-pharmacological techniques, with the highest rate of demonstration occurring once a month (34%), and one quarter often used once a week (25.5%).

**Table 3:** Distribution of the studied nurses according to nurses’ general pain practices.

Q	Nurses’ general pain practices	(N)	%
**15**	What are some non-pharmacological pain management therapies that you could use in the hospital without a doctor’s order? *		
	**Cognitive method**		
**1**	Preparatory information	0	0.0
**2**	Imagery	0	0.0
**3**	Distraction	32	68.1
**4**	Relaxation	17	36.2
**5**	Breathing technique	17	36.2
**6**	Positive reinforcement	25	53.2
	**Physical method**		
**7**	Thermal regulation	10	21.3
**8**	Message	14	29.8
**9**	Positioning	8	17.0
**10**	**Emotional support**	44	93.6
**11**	**Comfortable environment**	8	17.0
**12**	**Patient–family involvement**	0	0.0
**16**	**How often do you use any of the above therapies?**		
	Every day	6	12.8
	At least 3 times a week	4	8.5
	Once a week	12	25.5
	Once every other week	2	4.3
	Once a month	16	34.0
	Never	7	14.9

Table 4 displays nurses’ perception regarding the application of non-pharmacological pain management to reduce patients’ pain as the cognitive-behavioral methods, imagery, distraction technique, and positive reinforcement recorded the highest scores, whereas the nurses like to demonstrate the physical methods as the positioning, massage, and thermal regulation (91.1%, 87.2%, and 72.3% respectively). Meanwhile, the levels of comfort, patient family involvement, and emotional support (91.4%, 87.2%, and 83.0%, respectively) were all potentially applicable. Notably, the overall distribution of preferable non-pharmacological pain management methods among the study sample is identified as: comfortable environment, cognitive-behavioral methods, emotional support, patient family involvement, and physical methods (3.34+0.89, 3.24+0.62, 3.24+0.79, 3.16+0.81, and 2.99+0.77).

**Table 4:** Nurses’ perception of applicable non-pharmacological methods that reduce their patient’s pain items.

	No		Yes		Mean ± SD
	No	%	No	%	
**Cognitive-behavioral methods**					**3.24 ± 0.62**
Preparatory information	9	19.1	38	80.9	3.35 ± 0.85
Imagery	6	12.8	41	87.2	3.09 ± 0.51
Distraction technique	6	12.8	41	87.2	3.28 ± 0.46
Relaxation technique	10	21.2	37	78.7	3.04 ± 0.98
Breathing technique	10	21.3	37	78.8	3.26 ± 1.03
Positive reinforcement	6	12.8	41	87.2	3.49 ± 0.98
**Physical methods**					**2.99 ± 0.77**
Thermal regulation (Cold /Heat)	13	27.7	34	72.3	2.68 ± 0.89
Message	6	12.8	41	87.2	3.32 ± 1.02
Positioning	4	8.6	43	91.4	3.28 ± 0.88
**Emotional support**	8	17.0	39	83.0	**3.24 ± 0.79**
**Comfortable environment**	4	8.6	43	91.4	**3.34 ± 0.89**
**Patient family involvement**	6	12.8	41	87.2	**3.16 ± 0.81**

The relations between non-pharmacological technique scores and nurses’ demographic characteristics are shown in "Table 5". Results showed that male gender had a statistically significant association with the cognitive-behavioral methods, patient family involvement, and overall non-pharmacological methods, t (p) 2.324 (0.025), 2.177 (0.035), and 2.315 (0.025). Besides, the age group from 30–39 had the highest mean score of 66.38 ± 11.82 and was statistically significant with physical methods, F (p) 3.462 (0.040). However, the level of education of the nurses with technical institute level was statistically significant with cognitive-behavioral methods, whereas the baccalaureate level had the highest mean score in utilizing physical methods and, overall, non-pharmacological methods, F (p) 3.241 (0.031), 4.388 (0.009) and 3.379 (0.027).

**Table 5:** Relationship between demographic characteristics and non-pharmacological methods.

Demographic Questionnaire	Cognitive-behavioral methods	Physical methods	Emotional support	Comfortable environment	Patient family involvement	Overall non pharmacological methods
	Mean ± SD	Mean ± SD	Mean ± SD	Mean ± SD	Mean ± SD	Mean ± SD
**Sex**						
Male	66.38 ± 11.82	59.03 ± 16.27	67.59 ± 18.84	64.81 ± 13.36	66.67 ± 17.68	65.59 ± 11.54
Female	53.67 ± 15.30	47.53 ± 19.47	53.29 ± 19.33	53.73 ± 19.78	50.99 ± 19.79	52.95 ± 15.34
**t (p)**	2.324*(0.025*)	1.637(0.109)	2.005(0.051)	1.591(0.119)	2.177*(0.035*)	2.315*(0.025*)
**Age range (years)**						
20–29	52.70 ± 17.12	44.76 ± 20.67	54.03 ± 21.50	52.42 ± 22.17	50.81 ± 22.11	51.91 ± 17.13
30–39	61.21 ± 7.55	60.80 ± 14.00	62.12 ± 19.14	64.02 ± 9.18	63.64 ± 16.25	61.73 ± 9.19
40–49	66.03 ± 10.64	56.25 ± 0.00	55.00 ± 4.56	59.17 ± 3.49	52.50 ± 5.59	62.84 ± 7.01
**F(p)**	2.553(0.089)	3.462*(0.040*)	0.671(0.516)	1.622(0.209)	1.702(0.194)	2.448(0.098)
**Level of education**						
Diploma	51.07 ± 22.50	37.89 ± 26.07	51.04 ± 28.03	47.14 ± 29.02	51.56 ± 28.82	47.59 ± 20.84
Technical institute	60.22 ± 8.82	56.88 ± 8.09	57.50 ± 10.78	60.62 ± 4.96	56.25 ± 9.51	59.60 ± 6.99
Baccalaureate	59.63 ± 8.31	60.42 ± 13.50	65.28 ± 17.01	66.67 ± 14.67	64.58 ± 20.03	65.81 ± 12.71
**F(p)**	3.241*(0.031*)	4.388*(0.009*)	0.800(0.501)	2.461(0.075)	1.564(0.212)	3.379*(0.027*)
**Years of experience**						
0–9	54.82 ± 16.34	47.98 ± 20.86	56.62 ± 21.30	54.41 ± 21.76	54.04 ± 21.70	54.20 ± 16.61
10–19	63.45 ± 6.75	62.50 ± 15.31	63.33 ± 20.92	67.50 ± 4.56	67.50 ± 11.18	64.09 ± 7.98
20–29	57.00 ± 15.29	49.22 ± 11.30	48.96 ± 10.39	54.69 ± 6.84	45.31 ± 13.26	54.90 ± 12.77
**F(p)**	0.689(0.508)	1.253(0.296)	0.854(0.433)	1.042(0.361)	1.930(0.157)	0.898(0.415)

t: Student t-test F: F for ANOVA test

*: Statistically significant at p ≤ 0.05

Table 6 shows the univariate linear regression model for the parameters affecting overall nonpharmacological methods. It demonstrates that the statistically significant independent parameters that influenced overall nonpharmacological methods were male sex, who had a high score in their perception of applying non-pharmacological pain management therapies (B = -12.646, P = 0.025), whereas older nurses had less knowledge of implementing various non-pharmacological pain management methods (B = 6.691, P = 0.042), as well as the level of education, played a key role were nurses who were the holder of Baccalaureate certificate capable to apply non-pharmacological pain management therapies (B = 6.954, P = 0.008).

**Table 6:** Univariate linear regression analysis for the parameters affecting overall nonpharmacological methods.

Parameters	Univariate	
	p	B (95%C.I)
**Sex**	0.025*	-12.646 (-23.648 –-1.645)
** Male (65.59 ± 11.54)**		
Female (52.95 ± 15.34)		
**Age range (years)**	0.042*	6.691(0.244–13.138)
20–29 (51.91 ± 17.13)		
30–39 (61.73 ± 9.19)		
** 40–49 (62.84 ± 7.01)**		
**Level of education**	0.008*	6.954(-11.961– -1.947)
Diploma (47.59 ± 20.84)		
Technical institute (59.60 ± 6.99)		
** Baccalaureate (65.81 ± 12.71)**		
**Years of Experience**	0.661	1.308(-4.653–7.269)
0–9 (54.20 ± 16.61)		
10–19 (64.09 ± 7.98)		
20–29 (54.90 ± 12.77)		

R^2^ = 0.214, F = 3.906*, p = 0.015*

B: Unstandardized Coefficients

C.I: Confidence interval LL: Lower limit UL: Upper Limit

#: All variables with p<0.05 was included in the multivariate

*: Statistically significant at p ≤ 0.05

**Fig 1:**
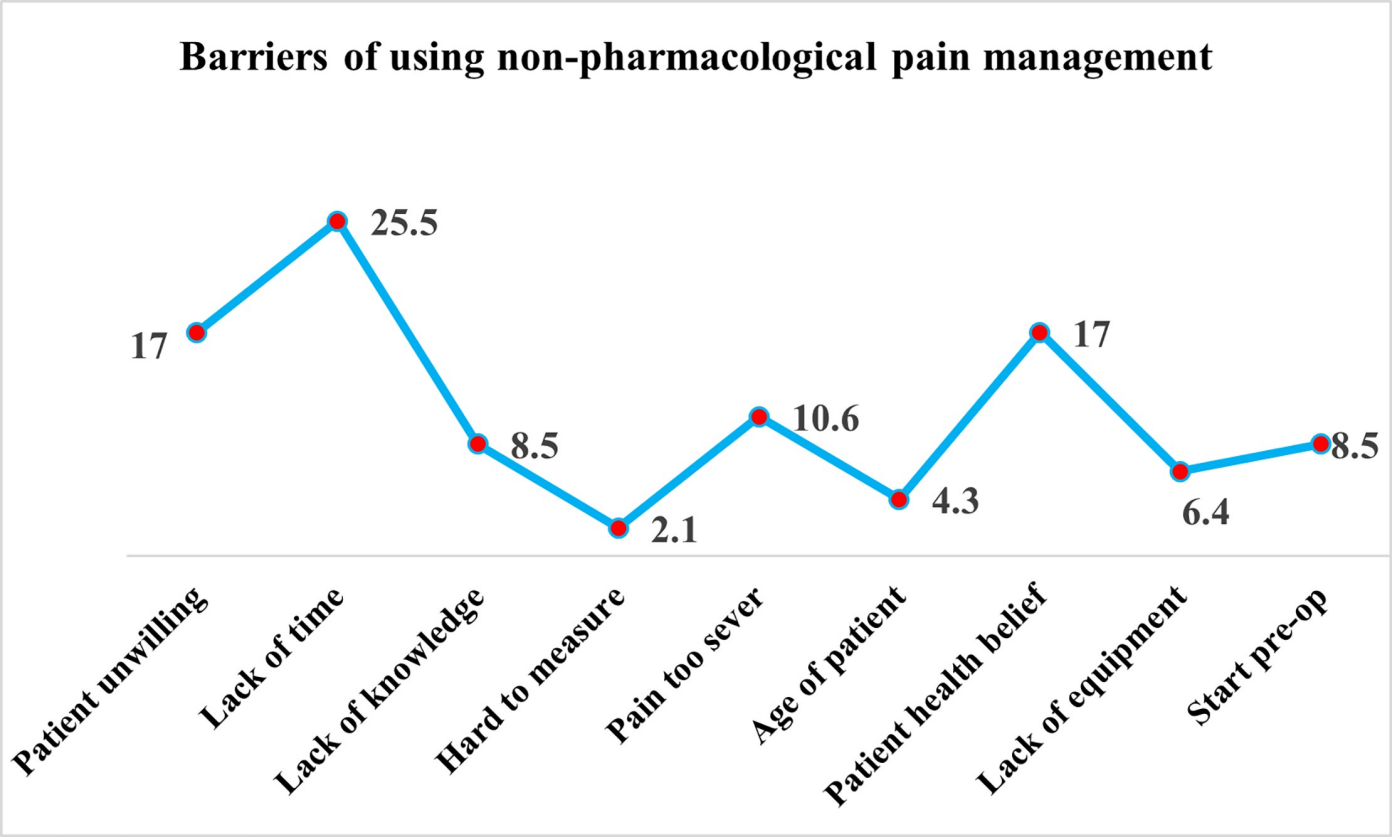
Barriers of using non-pharmacological pain management.

Fig 1 displays nurses’ perception regarding the barriers to using non-pharmacological pain management Nurses reported that the highest barrier to applying nonpharmacological pain management was lack of time (25.5%), followed by patient unwillingness (17%) and patient health beliefs (17%).

**Fig 2:**
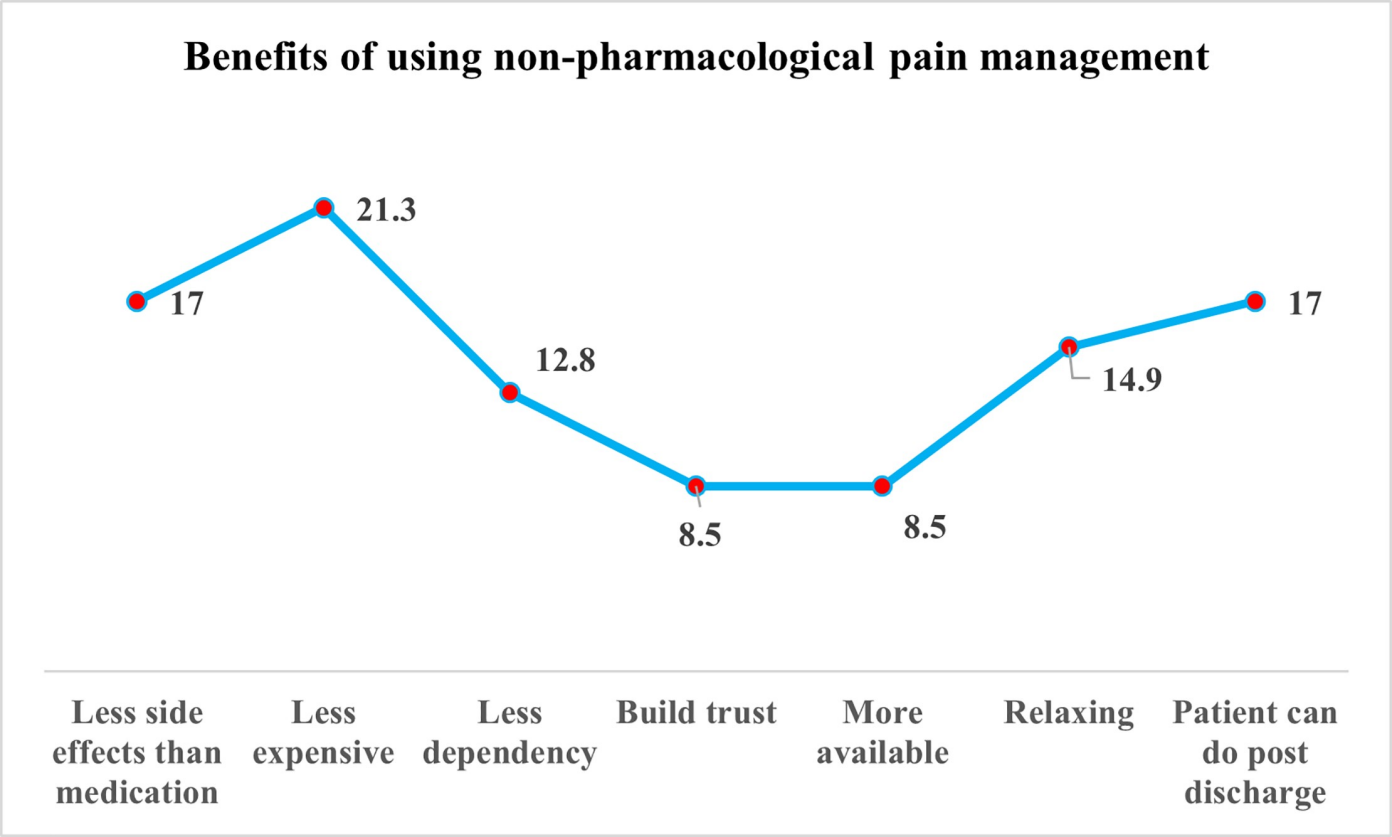
Benefits of using non-pharmacological pain management.

Nevertheless, results showed that most of the nurses explored different aspects of the benefits of using nonpharmacological pain management techniques (Fig 2). Results indicated that nurses recognized the nonpharmacological pain management techniques as less expensive (21%), with fewer side effects than medication (17%), the patient can demonstrate post-discharge (17%) and can be used as a relaxation technique (14.9%).

Implementation of effective non-pharmacological management requires knowledgeable, skillful nurses. Many studies have shown that nurses require more knowledge about non-pharmacological management to reduce patients’ pain intensity [17–19].

The present study was carried out on a convenience sample of 47 nurses. Although not randomly selected, their demographic characteristics are similar to those reported in a study by Adams et al. 2020, the mean age and years of experience, as well as the majority of the participants were females and they had not received training sessions on pain management [19].

Considering pain as a fifth vital sign, for instance, regular pain assessment is recommended by using an available pain assessment tool. As reported in the present study, the pain assessment was not adhered to by nurses and was not used for pain assessment as supported by Kiwanuka & Masaba, 2018 [20]. In agreement with Ung et al, 2016 who have emphasized the importance of assessing pain, their study recognized a lack of pain assessment among nurses [21]. Moreover, the authors highlight that the pain assessment is a key toward alleviating the pain. This is incongruent with Van den Beuken-van Everdingen et al, 2007, who have reported that nurses have adequate knowledge of assessing and managing pain [22].

Nurse’s perception regarding applying non-pharmacological techniques without doctor order was admitted in the present study, in congruence with many studies that addressed the distraction techniques are doable and have the greatest benefit on the patient with anxiety [9–12].

Interestingly, the current study findings about preferred nonpharmacological pain management techniques among nurses support results of Kidanemariam et al., 2020, who highlighted that among cognitive-behavioral methods, breathing techniques, relaxation, (81.7% and 72.1%), for physical positioning, followed by thermal regulation, heat/cold, and massage (84.4%, 23.4%, and 18.8%), whereas emotional support was reported as the highest score (92.2%) [23].

Positioning is considered one of the highest methods that nurses can utilize to alleviate pain, as most applicable physical methods of non-pharmacological pain management are integrated into routine nursing intervention during the postoperative phase, although thermal regulation and massage application are used at a minimum level [15, 24, 25].

A similar study has been done by Gelinas et al, 2013, who have evaluated the patient’s and nurses’ perspectives of non-pharmacological pain management and reported that the massaging therapy as physical nonpharmacological pain management was among critically ill patients was effective [26].

A recent study conducted in Ghana to evaluate the nurses’ knowledge and attitude towards postoperative pain management and found there was no significant relationship between nurses’ knowledge and years of experience or number of years of working in the surgical ward, which quietly close to the present study findings [19].

A single study was conducted in 1999 by Salanterä et al, who have highlighted the important role of knowledgeable nurses to apply non-pharmacological pain management techniques. Based on that, many studies have been done in different countries and have emphasized assessing nurses’ attitudes regarding utilization of pain management in the United States of America (USA) [27], Hong Kong, China, and have reported that nurses have an adequate level of attitude [28]. Meanwhile, Muwanza et al 2019, studied the level of nurses’ knowledge and indicated that nurses have poor knowledge about nonpharmacological pain management, thus a supportive educational session is recommended [29]. In addition, a recent study addressed those nurses who received continuous education sessions had a positive attitude toward applying nonpharmacological pain management therapies [14].

Regarding the level of education, the present study findings are in accordance with Kahsay et al, 2019, who found that those with baccalaureate degrees are more likely to utilize nonpharmacological pain management therapies as compared to those who have diplomas [18]. Likewise, in similar studies done in Egypt, Saudi Arabia, Jordan, India, Uganda, and the western region [27, 30–32].

Nurses in the present study identify many barriers to applying nonpharmacological pain management in the agreement of Zahed Pasha et al, 2017 who have similar barriers as lack of time and overloaded nurses (2.0±35.6) [33]. Also, and in congruence with the present study findings, Becker et al, 2017 have reported that the barriers to demonstrate non-pharmacological pain management therapies are patients unwilling or unmotivated to apply and patients’ beliefs that medication is more effective to relieve pain than non-pharmacological therapies [34]. Simultaneously, nurses embedded the benefits of using non-pharmacological techniques, in line with the present study findings. Goštautaitė et al, 2017, have emphasized that the implementation of non-pharmacological pain management therapy can eliminate the prescribing of pharmacological pain management as well as reduce the physiological and psychological consequences [35]. Moreover, a recent study highlighted another benefit, reducing pain intensity, increasing quality of life, increasing patients’ relaxation, and reducing the length of stay at the hospital [36].

Traditionally, pharmacological pain management therapy has been the best choice for managing pain, accompanied by unlikely side effects physically, cognitively, and economically [37]. Therefore, nurses considered that non-pharmacological pain management should be applied when patients had less pain intensity [38].

Nurses have a crucial role in providing pain management measures in surgical ward, and utilizing non-pharmacological pain techniques to improve patients’ outcomes [39].

## Conclusions

The insight gained from this study suggests that non-pharmacological pain management therapies have a valuable effect in managing moderate to mild pain intensity, especially if demonstrated in the preoperative phase. Nurses play a key role in applying different non-pharmacological therapies effectively based on their perception and application of cognitive-behavioral methods such as the distraction technique and positive reinforcement, whereas nurses like to demonstrate the physical methods of positioning, massage, and thermal regulation. Nurses expressed some of barriers that hinder the utilization of nonpharmacological pain management such as lack of time, patient unwillingness, and patients’ health beliefs. Nevertheless, non-pharmacological pain management is less expensive, and has fewer side effects than medication and the patient can demonstrate post-discharge. There is meticulous need for nurses to consider the application of non-pharmacological pain management therapies to patients undergoing surgical procedures to improve the quality of care, reduce undesired sedation side effects, and minimize the cost.

### Limitation

Limitation of the present study were:

1. This study adopted non-probability convenience sampling, and the study sample size is limited which may limit the generalization of finding beyond the study sample.
2. There could be a possibly of unequal responses related to the difference numbers between male and female nurses.
3. Cross-sectional research is measuring the outcome in the study at the same time and the data was obtained based on nurses’ response which may cause some biased responses.

## References

[1] American Pain Society. Principles of analgesic use in the treatment of acute and cancer pain (6^th^ ed.). Glenview, IL, 2008.

[2] Schug SA, Lavand’homme P, Barke A, Korwisi B, Rief W, Treede RD; IASP Taskforce for the Classification of Chronic Pain. The IASP classification of chronic pain for ICD-11: chronic postsurgical or posttraumatic pain. Pain. 2019 Jan;160(1):45–52. doi: 10.1097/j.pain.0000000000001413 30586070

[3] Pasero C., McCaffery M. Pain assessment and pharmacologic management. St. Louis, MO: Mosby-Elsevier, 2011.

[4] Pasero C., Puntillo K., Li D., Mularski RA., Grap MJ., Erstad BL., et al. (2009). Structured approaches to pain management in the ICU. Chest;135(6):1665–1672. doi: 10.1378/chest.08-2333 . 19497902

[5] Lehne R. A. Pharmacology for nursing care (8th ed.). St. Louis, MO: Elsevier Saunders, 2013.

[6] Chlan L, Halm MA. Does music ease pain and anxiety in the critically ill? Am J Crit Care. 2013;22(6):528–32. doi: 10.4037/ajcc2013998 . 24186825

[7] Hinkle J., & Cheever K. Brunner & Suddarth’s Textbook of Medical—Surgical Nursing. (14th ed.). Lippincott Williams& Wilkins, 2018.

[8] Gan TJ, Habib AS, Miller TE, White W, Apfelbaum JL. Incidence, patient satisfaction, and perceptions of post-surgical pain: results from a US national survey. Curr Med Res Opin. 2014;30(1):149–60. doi: 10.1185/03007995.2013.860019 24237004

[9] Dimitriou V, Mavridou P, Manataki A, Damigos D. The Use of Aromatherapy for Postoperative Pain Management: A Systematic Review of Randomized Controlled Trials. J Perianesth Nurs. 2017;32(6):530–541. doi: 10.1016/j.jopan.2016.12.003 29157760

[10] Poulsen MJ, Coto J. Nursing Music Protocol and Postoperative Pain. Pain Manag Nurs. 2018;19(2):172–176. doi: 10.1016/j.pmn.2017.09.003 29153918

[11] Cooley LF, Barker SB. Canine-assisted Therapy as an Adjunct Tool in the Care of the Surgical Patient: A Literature Review and Opportunity for Research. Altern Ther Health Med. 2018;24(3):48–51. . 29477136

[12] Mosso Vázquez JL, Mosso Lara D, Mosso Lara JL, Miller I, Wiederhold MD, Wiederhold BK. Pain Distraction During Ambulatory Surgery: Virtual Reality and Mobile Devices. Cyberpsychol Behav Soc Netw. 2019 Jan;22(1):15–21. doi: 10.1089/cyber.2017.0714 Epub 2018 Sep 25. 30256662

[13] Brennan F, Carr DB, Cousins M. Pain management: a fundamental human right. Anesth Analg. 2007 Jul;105(1):205–21. doi: 10.1213/01.ane.0000268145.52345.55 . 17578977

[14] Jira L, Weyessa N, Mulatu S, Alemayehu A. Knowledge and Attitude Towards Non-Pharmacological Pain Management and Associated Factors Among Nurses Working in Benishangul Gumuz Regional State Hospitals in Western Ethiopia, 2018. J Pain Res. 2020; 16;13:2917–2927. doi: 10.2147/JPR.S265544 ; PMCID: PMC7678465. 33235490 PMC7678465

[15] Pölkki T, Vehviläinen-Julkunen K, Pietilä AM. Nonpharmacological methods in relieving children’s postoperative pain: a survey on hospital nurses in Finland. J Adv Nurs. 2001;34(4):483–92. doi: 10.1046/j.1365-2648.2001.01777.x . 11380715

[16] Bicek, E. Nurses’ Attitudes, Knowledge, and Use of Nonpharmalogical Pain Management Techniques and Therapies. Honors Projects.2004; Paper 12. Retrieved from https://digitalcommons.iwu.edu/nursing_honproj/12/

[17] Albaqawi H., Maude P., Shawhan-Akl L. Saudi Arabian Nurses’ Knowledge and Attitudes Regarding Pain Management: Survey Results Using the KASRP. International Journal of Health Sciences & Research. 2016; 6 (12), 150–164. https://www.ijhsr.org/IJHSR_Vol.6_Issue.12_Dec2016/24.pdf.

[18] Kahsay DT, Pitkäjärvi M. Emergency nurses´ knowledge, attitude and perceived barriers regarding pain Management in Resource-Limited Settings: cross-sectional study. BMC Nurs. 2019 Nov 21;18:56. doi: 10.1186/s12912-019-0380-9 . 31832015 PMC6873521

[19] Adams SM, Varaei S, Jalalinia F. Nurses’ Knowledge and Attitude towards Postoperative Pain Management in Ghana. Pain Res Manag. 2020 Aug 7;2020:4893707. doi: 10.1155/2020/4893707 . 32831982 PMC7429762

[20] Kiwanuka F., Masaba R. Nurses’ knowledge, attitude and practices regarding pain assessment among patients with cancer at Uganda Cancer Institute. Journal of Research in Clinical Medicine. 2018; 6(2), 72–9. doi: 10.15171/jarcm.2018.011

[21] Ung A, Salamonson Y, Hu W, Gallego G. Assessing knowledge, perceptions and attitudes to pain management among medical and nursing students: a review of the literature. Br J Pain. 2016;10(1):8–21. doi: 10.1177/2049463715583142 27551407 PMC4977961

[22] Van den Beuken-van Everdingen MH, de Rijke JM, Kessels AG, Schouten HC, van Kleef M, Patijn J. Prevalence of pain in patients with cancer: a systematic review of the past 40 years. Ann Oncol. 2007;18(9):1437–49. doi: 10.1093/annonc/mdm056 17355955

[23] Kidanemariam BY, Elsholz T, Simel LL, Tesfamariam EH, Andemeskel YM. Utilization of non-pharmacological methods and the perceived barriers for adult postoperative pain management by the nurses at selected National Hospitals in Asmara, Eritrea. BMC Nurs. 2020; 22,19:100. doi: 10.1186/s12912-020-00492-0 33110397 PMC7583254

[24] He HG, Pölkki T, Vehviläinen-Julkunen K, Pietilä AM. Chinese nurses’ use of non-pharmacological methods in children’s postoperative pain relief. J Adv Nurs. 2005; 51(4):335–42. doi: 10.1111/j.1365-2648.2005.03505.x . 16086802

[25] He HG, Jahja R, Lee TL, Ang EN, Sinnappan R, Vehviläinen-Julkunen K, et al. Nurses’ use of non-pharmacological methods in children’s postoperative pain management: educational intervention study. J Adv Nurs. 2010;66(11):2398–409. doi: 10.1111/j.1365-2648.2010.05402.x 20722797

[26] Gélinas C, Arbour C, Michaud C, Robar L, Côté J. Patients and ICU nurses’ perspectives of non-pharmacological interventions for pain management. Nurs Crit Care. 2013;18(6):307–18. doi: 10.1111/j.1478-5153.2012.00531.x 24165072

[27] Salanterä S, Lauri S, Salmi TT, Helenius H. Nurses’ knowledge about pharmacological and nonpharmacological pain management in children. J Pain Symptom Manage. 1999; 18(4):289–99. doi: 10.1016/s0885-3924(99)00065-2 10534969

[28] Tse MM, Chan BS. Knowledge and attitudes in pain management: Hong Kong nurses’ perspective. J Pain Palliat Care Pharmacother. 2004;18(1):47–58. . 15148008

[29] Mwanza E, Gwisai RD, Munemo C. Knowledge on Nonpharmacological Methods of Pain Management among Nurses at Bindura Hospital, Zimbabwe. Pain Res Treat. 2019, 1;2019:2703579. doi: 10.1155/2019/2703579 30693106 PMC6332921

[30] Kizza, IB. Nurses’ knowledge and practices related to pain assessment in critically ill patients at Mulago hospital, Uganda. Muhimbili University Health Allied Sciences. 2012; https://core.ac.uk/download/pdf/11307864.pdf.

[31] Ali S, Ibrahim Y., Mohamed E. Non-pharmacological pain management: nurses’ knowledge, attitudes and practices in selected hospitals at Makkah El-Mukarramah. Life Science Journal. 2013;10(2):1327–1335.http://www.lifesciencesite.com/lsj/life.

[32] Badr MN., Morsy WY., Ali NS. Critical care nurses’ knowledge and practices regarding Pain assessment and management at Cairo University Hospitals. Egyptian Nursing Journal.2015;10(1):28–38. file:///C:/Users/HP/Downloads/4223-8275-1-SM.pdf.

[33] Zahed Pasha Y., Arzani A., Akbariyan Z., Ahmadi M. Barriers to Use of Non pharmacological Pain Management Methods in Neonatal Intensive Care Unit. J Babol Univ Med Sci. 2017;19(9):20–5. http://jbums.org/files/site1/user_files_a248ba/eng/ahmadi_-A-10-2787-1-4727155.pdf.

[34] Becker W., Dorflinger L., Edmond S., Islam L., Heapy A., Fraenkel L. (2017). Barriers and facilitators to use of nonpharmacological treatments in chronic pain. Becker et al. BMC Family Practice 20:18–41. doi: 10.1186/s12875-017-0608-2 PMC5359906 28320337

[35] Goštautaitė S., Piščalkienė V., Sari Laanterä S., Uosukainen L. (2017). Non—Pharmacological Pain Management In Postoperative Care Of School—Age Children. Visuomenės Sveikata 27(6):71–79. dio: doi: 10.5200/sm-hs.2017.099

[36] Yaban Z. (2019). Usage of Non-Pharmacologic Methods on Postoperative Pain Management by Nurses: Sample of Turkey. International Journal of Caring Sciences January (12).1:529–540.

[37] Karabulut N. (2016). Non-pharmacological interventions for pain manage ment used by nursing students in Turkey. Journal of Nursing and Social Sciences. 1:23–31.10. dio: doi: 10.1016/j.kontakt.2015.12.00

[38] Thomas E., Weiss S. (2000). Nonpharmacological interventions with chronic cancer pain in adults. Cancer Control, (7),2:157–164. doi: 10.1177/107327480000700206 10783820

[39] Voshall B., Dunn KS., Shelestak D. (2013). Original article knowledge and attitudes of pain management among nursing faculty. Pain Manag Nurs. 14:e226–e235. doi: 10.1016/j.pmn.2012.02.001 24315276

